# Lipid nanoparticle‐mediated RNA delivery for immune cell modulation

**DOI:** 10.1002/eji.202451008

**Published:** 2024-09-16

**Authors:** Emily H. Kim, Sridatta V. Teerdhala, Marshall S. Padilla, Ryann A. Joseph, Jacqueline J. Li, Rebecca M. Haley, Michael J. Mitchell

**Affiliations:** ^1^ Department of Bioengineering School of Engineering and Applied Science University of Pennsylvania Philadelphia Pennsylvania USA; ^2^ Abramson Cancer Center Perelman School of Medicine University of Pennsylvania Philadelphia Pennsylvania USA; ^3^ Center for Cellular Immunotherapies Perelman School of Medicine University of Pennsylvania Philadelphia Pennsylvania USA; ^4^ Penn Institute for RNA Innovation Perelman School of Medicine University of Pennsylvania Philadelphia Pennsylvania USA; ^5^ Institute for Immunology Perelman School of Medicine University of Pennsylvania Philadelphia Pennsylvania USA; ^6^ Cardiovascular Institute Perelman School of Medicine University of Pennsylvania Philadelphia Pennsylvania USA; ^7^ Institute for Regenerative Medicine Perelman School of Medicine University of Pennsylvania Philadelphia Pennsylvania USA

**Keywords:** Dendritic cells, Lipid nanoparticles, Macrophages, NK cells, T cells

## Abstract

Lipid nanoparticles (LNPs) have emerged as the preeminent nonviral drug delivery vehicles for nucleic acid therapeutics, as exemplified by their usage in the mRNA COVID‐19 vaccines. As a safe and highly modular delivery platform, LNPs are attractive for a wide range of applications. In addition to vaccines, LNPs are being utilized as platforms for other immunoengineering efforts, especially as cancer immunotherapies by modulating immune cells and their functionality via nucleic acid delivery. In this review, we focus on the methods and applications of LNP‐based immunotherapy in five cell types: T cells, NK cells, macrophages, stem cells, and dendritic cells. Each of these cell types has wide‐reaching applications in immunotherapy but comes with unique challenges and delivery barriers. By combining knowledge of immunology and nanotechnology, LNPs can be developed for improved immune cell targeting and transfection, ultimately working toward novel clinical therapeutics.

## Graphical abstract

A review of lipid nanoparticle development and application for engineering T cells, NK cells, macrophages, dendritic cells, and stem cells for immunotherapies.

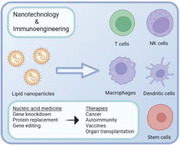

## Introduction

Immunotherapy is experiencing a renaissance, propelled by its convergence with nanotechnology [1]. The intersection between these fields has unlocked new frontiers, as exemplified by the development of mRNA vaccines, including those for COVID‐19, which leverages mRNA technology to train the immune system to protect the body from viruses [2, 3]. The successful administration of these vaccines to hundreds of millions of people around the world underscores the immense potential of nucleic acid‐based immunotherapy. Moreover, the recent Nobel Prize in Medicine to Dr. Karikó and Dr. Weissman, the founders of mRNA medicine, has further affirmed this significance. Sitting at the heart of this renaissance, nucleic acid‐based immunotherapy extends beyond vaccines to treat diseases including genetic disorders, autoimmune conditions, and cancer [4, 5]. By precisely directing cellular activities, nucleic acid therapies enable tailored interventions, offering a potential paradigm shift in medicine.

Nanotechnology has proved immensely valuable for the development of nucleic acid medicines as nucleic acid cargoes induce unwanted activation of the immune system and degrade rapidly before reaching their intended targets [6, 7]. However, with nanotechnology, nucleic acids can be encapsulated by protective carriers, ensuring safe delivery to the target cells or tissues. Moreover, the precise manipulation and design of nanoscale materials can enable controlled release, which enhances therapeutic efficacy while minimizing off‐target transfection [8]. The synergy between nanotechnology and nucleic acid‐based immunotherapies has opened the door for new targeted interventions.

A popular target for these therapies is immune cells. By precisely modulating immune cells, such as T cells, natural killer (NK) cells, dendritic cells, and others, nucleic acid‐based immunotherapies can deliver instructions to enhance their ability to fight cancer cells [9], improve their recognition of specific targets [10], or regulate their responses to prevent autoimmune reactions [11, 12]. For example, therapies can involve delivering mRNA to encode specific antigens for increasing recognition [13, 14, 15] or using siRNA to silence genes that impede immune activity [16, 17, 18]. This capacity to precisely modulate immune responses through nucleic acid technology presents a novel dimension for the development of targeted therapies.

**Figure 1:**
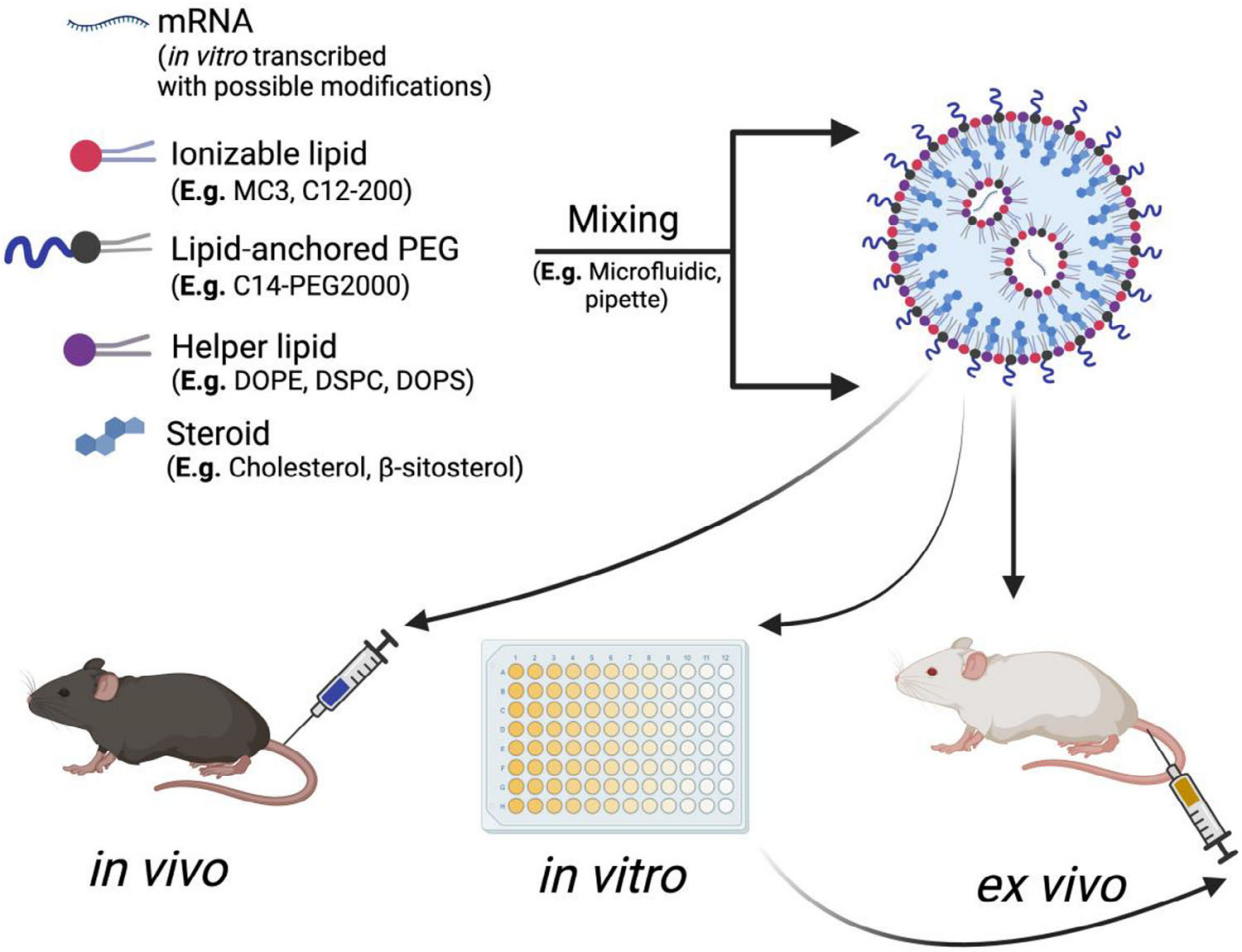
Overview of mRNA lipid nanoparticle (LNP) formulation. Excipients of LNPs include an ionizable lipid, a lipid‐anchored PEG, a helper lipid, and a steroid that is mixed with a nucleic acid of interest. The lipid phase and the nucleic acid phase are mixed, often through microfluidics or pipette mixing, to formulate the LNP. The LNP can then be administered *in vivo*, *in vitro* to transfect a cell line, or *ex vivo*, where cells are transfected apart from the body and then readministered. Created with BioRender.

Among the abundant strategies at the nanotechnology and immunotherapeutic intersection, the utilization of lipid nanoparticles (LNPs) stands as one of the most promising, being the delivery vehicle for the mRNA COVID‐19 vaccines [19]. LNPs protect nucleic acid cargo from degradation by nucleases and immune recognition via the lipid shell and help facilitate delivery directly to the organ, tissue, or cell of interest, simultaneously limiting off‐target delivery [20, 21]. LNPs have four main components: a cholesterol element for stability and improved membrane fusion, a helper phospholipid for aiding cargo encapsulation and endosomal escape, a lipid‐anchored polyethylene‐glycol conjugate to reduce LNP aggregation, and an ionizable lipid (Fig. 1) [22, 23]. To formulate LNPs, the lipid components are traditionally dissolved in an ethanolic phase, which is then mixed with an aqueous low‐pH buffer containing the nucleic acid.

The ionizable lipid plays an essential role in the efficacious delivery of LNPs due to its flexible ionic charge, where it maintains a neutral state under normal physiological conditions but becomes positively charged in acidic environments [24, 25]. Therefore, the p*K*_a_ of the ionizable lipid is crucial for the functionality of the LNP, especially considering that many cells have distinct endosomal–lysosomal pathways [26]. During formulation, the lipid and mRNA components are mixed at a low pH to allow electrostatic binding of the ionizable lipid and mRNA to promote encapsulation; however, upon dialysis, the ionizable lipids become neutral. This is important since upon entering the acidic endosomal–lysosomal pathway, the re‐protonated ionizable lipids disrupt the endosomal membranes and allow the cargo to escape before degradation [27]. The ζ‐potential of the LNP is an indicator of the surface charge and can affect the tropism of the overall LNP by binding to serum proteins with different ionic charge states [28].

LNPs offer flexibility in the structure of the core components, allowing for LNPs to be tailored for specific cell types and applications. For instance, the structure of lipids can be adjusted by altering the lipid tail length or by modifying functional groups on the ionizable lipid [29, 30]. Over the past decade, researchers have generated thousands of new lipid structures by employing high‐throughput synthetic techniques [31, 32]. Additionally, the type and ratio of lipid excipients can be easily modulated to enhance the potency of the LNPs depending on the cargo. For example, studies have demonstrated that adding 1,2‐dioleoyl‐sn‐glycero‐3‐phosphoethanolamine and increasing the ionizable lipid:mRNA weight ratio increases the potency of mRNA‐loaded LNPs [33]. Finally, a fifth component — or even multiple components — can be incorporated into the LNPs to enhance their efficacy or to target organs, tissue, and cell types of interest more selectively. This can include adding a permanently positively charged lipid such as 1,2‐dioleoyl‐3‐trimethylammonium‐propane, which has been shown to favor lung delivery, whereas adding a negatively charged lipid, like 1,2‐distearoyl‐sn‐glycero‐3‐phosphate, can lead to spleen delivery [34].

**Figure 2:**
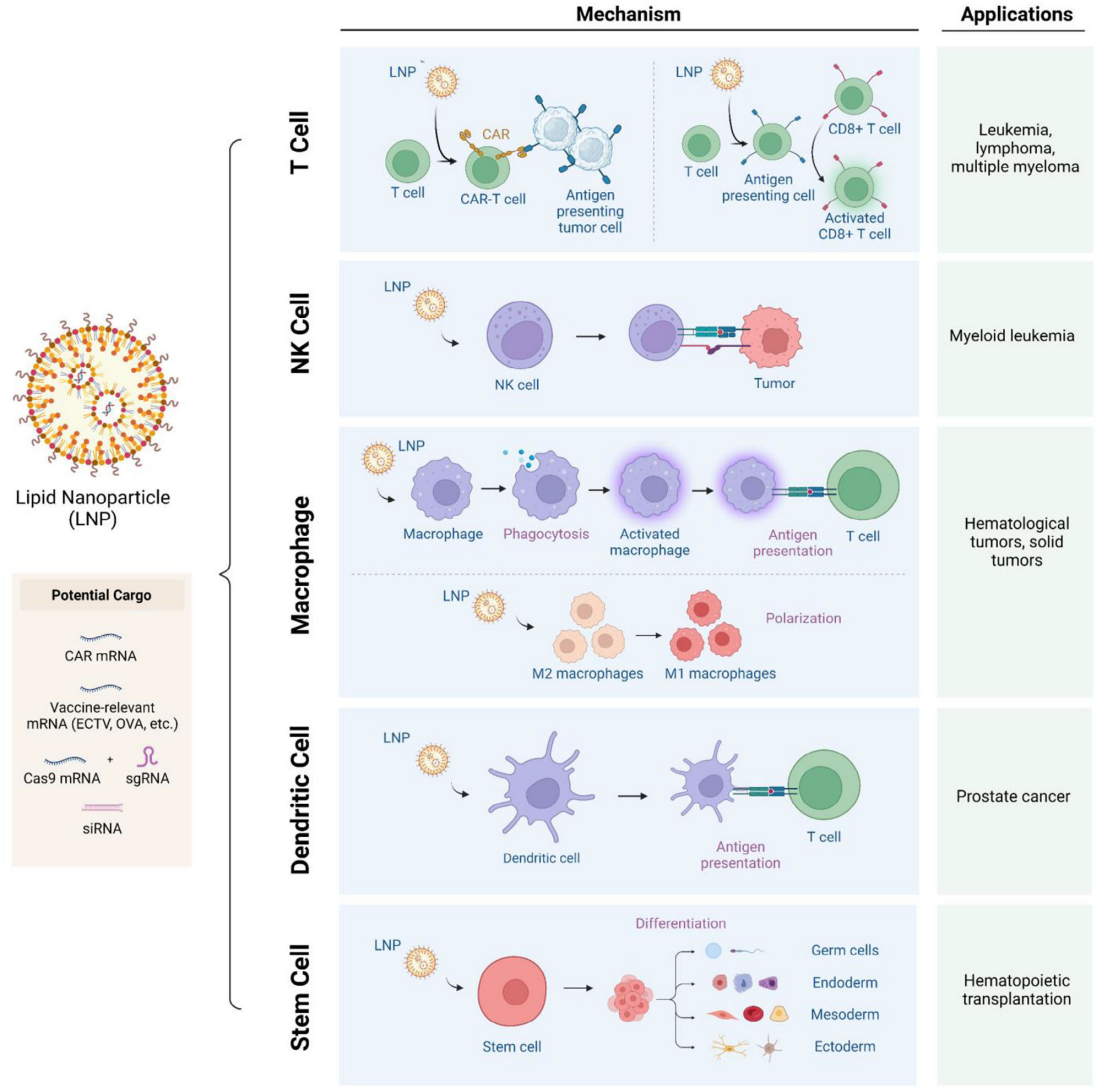
Schematic of lipid nanoparticle (LNP) applications for T cells, NK cells, macrophages, stem cells, and dendritic cells. The LNPs can be encapsulated with a variety of functional cargo including gene editing materials and antigens (left). Depending on the application, the LNP‐based nucleic acid therapy can activate immune cells, facilitate differentiation, repolarize, and install targeting moieties (middle). List of specific applications involving modulating immune cells with LNPs (right) [35, 36, 37, 38, 39]. Created with BioRender.

The tunability, low toxicity, and potency of LNPs make them a viable option for nucleic acid delivery for the development of immunotherapies. Here, we discuss the current progress in employing LNPs for modulating immune cells for a variety of applications and consider the future direction for LNP‐enabled immunotherapies. A summary of the LNP‐based approaches in each cell type of interest can be found in Fig. 2.

## T cells

T lymphocytes, or T cells, are a type of leukocyte that is crucial to the operation of the immune system. There are many types of T cells, including helper T cells (CD4^+^ T cells), cytotoxic T cells (CD8^+^ T cells), and regulatory T cells (T regs) that have distinct functions in impacting the adaptive immune response [40]. The synergistic actions of each type of T cell can protect the host from viral infections, pathogens, and other foreign or intrinsically aberrant species. For instance, CD4+ T cells recognize antigen‐presenting cells (APCs) and recruit other immune cells, such as CD8+ T cells, to facilitate eliminating unhealthy cells. T cells play a crucial role in coordinating the immune system and eradicating diseases, and thus, this primes them as an ideal target for immunomodulatory medicine and has led to a strong focus in recent years on T cells as autologous cellular immunotherapy.

Chimeric antigen receptor (CAR) T cell therapies are FDA‐approved and effective treatments for combatting cancer, especially blood cancers, including multiple myeloma [41], acute myeloid leukemia [42], and Hodgkin's lymphoma [43], with a total of six CAR T cell treatments approved to date [44]. CAR receptors are engineered to recognize and bind to specific antigens on the surface of cancer cells and consist of three components: an antigen‐binding domain, a transmembrane domain, and an intracellular signaling domain that can direct lymphocytes to identify and eradicate the specific ligand that the CAR targets. This makes the therapy highly translatable to different types of cancers and immune cell targets, as it can be programmed to target different clusters of differentiation (CDs). One of the most prevalent types of CAR T cells is anti‐CD19 (aCD19) CAR T cells, which attack B cell lymphomas by presenting a human‐CD19 receptor. The therapy has traditionally been applied *ex vivo* to engineer a patient's own T cells to permanently express the receptor, which subsequently is re‐injected into the patient to eradicate malignant CD19^+^ B cells. Lentiviral transduction is the primary modality in which T cells are converted to CAR T cells, where a viral vector, most commonly derived from HIV‐1, is used to integrate the CAR gene into the cell's genome [45]. Electroporation, where a patient's cells are electrified to create pores to allow exogenous material to enter, is also employed to create CAR T cells. EP is effective in transfecting cells but has low throughput and can damage the cells. These methodologies also induce permanent expression of CAR, which can lead to patients suffering from cytokine storms, macrophage activation syndrome, and neurotoxicity [46].

A promising alternative to permanent CAR expression is nucleic acid‐based therapeutics such as CAR mRNA. mRNA therapeutics are advantageous because there is no risk of genomic integration and they exhibit lower immunogenicity than plasmids. Delivery of CAR mRNA also results in transient CAR expression, which reduces the effects seen with permanent expression of CAR by preventing the development of a large‐scale positive feedback loop of cytokine release by millions of CAR T cells that induce cytokine storms [47]. Several studies have been conducted within the past five years exploring the ability to deliver CAR mRNA to T cells by utilizing LNPs [48]. Additionally, LNPs provide a beneficial alternative to viral vectors due to their standing FDA approval and lower cost of production [49]. CAR mRNA LNPs have been shown *in vitro* to transform T cells into CAR T cells, and the killing activity of LNP‐derived aCD19 CAR T cells was seen to be comparable to those of EP‐derived aCD19 CAR‐T cells [50]. This presents a promising alternative to more harmful methodologies when using CAR T cells to attack B lymphomas and other cancers. Further, LNP‐generated CAR T cells have higher retention of viability than EP‐created CAR T cells due to the transmission of CAR mRNA by endocytosis versus electroporated cells [51] For example, in a recent study comparing the efficacy and cytotoxicity of EP‐derived CAR T cells versus LNP‐derived CAR T cells, electroporation of the primary T cells resulted in higher apoptosis and cell death (29%) than LNP treated primary T cells (16%) [52].

The successful delivery, transfection, and efficacy of CAR mRNA is heavily contingent on both the LNP and the mRNA. Our group has explored the importance of the lipid components of the LNP. First, we optimized the ionizable lipid for delivery to patient‐derived T cells, identifying C14‐4 as a lead candidate for potent delivery of CD19‐CAR mRNA [53]. In the following project, we optimized the excipient molar ratios of the LNP including increasing the ionizable lipid and helper lipid ratios by decreasing the cholesterol molar ratio. From this, an optimized lipid nanoparticle was found to transfect T cells more effectively with C14‐4 as the ionizable lipid [54].

Second, the mRNA itself can be optimized to reduce immunogenicity and increase the overall translated protein through chemical nucleoside modifications. Multiple studies have demonstrated that nucleoside modifications, the editing of the nitrogenous base of a specific nucleotide, have shown greater efficacy and lower immunogenicity [55, 56]. The modification with the highest efficacy is the inclusion of N1‐methylpseudouridine in place of uracil, where this modification was shown to increase mRNA expression in many cell lines [57]. Additionally, these modifications reduce the targeting of the exogenous mRNA and allow the mRNA to pass through the T cells relatively undetected by cell receptors and nucleases. These modifications, and their resulting effect on immunogenicity, have played a role in the possible expansion of CART‐cell therapy from solely *ex vivo* to an *in vivo* application. By producing CART cells *in vivo*, the overall therapy process could be simplified and accelerated, if effective delivery can be reliably achieved with limited side effects.

Researchers have explored alternative ways to elicit endogenous CAR T cell expression, mainly via viral vectors delivering CAR plasmids. This, however, also introduces a strong immune response and runs the risk of random insertion into the genome, which may cause secondary effects, such as mutations in off‐target genes that further disrupt cellular functions [58]. Recently, CAR T cells have been created via LNPs for *in vivo* applications aside from cancer immunotherapies, such as cardiac fibrosis. Experiments by Rurik et al. [59] generated CAR T cells through LNPs that were antibody‐conjugated to specifically target CD5^+^ T cells that recognize fibroblast activation protein (FAP) in cardiac fibrosis, which is a disease of the heart that results in the stiffening and scarring of heart tissue. The resulting aFAP CAR CD5^+^ T cells were able to target fibroblasts *in vivo*, killing scarred heart tissue. This alternative use of CAR demonstrates the versatility of its applications within the field and its further promise.

LNPs provide a viable route for T cell‐related activities and therapies that offer a mechanism for immune system activation and modulation and there are advantages to delivering mRNA to T cells to perform other immunogenic activities beyond CAR T cell therapy. Mainly, this is through the use of LNPs as mRNA vaccines, the most notable example being for COVID‐19, which delivers SARS‐CoV‐2 mRNA to APCs [60]. In LNP‐mRNA vaccines, a specific antigen is introduced via the delivery of mRNA encoding for the spike protein to both immune cells and nonimmune cells at the injection site, which allows T cells to recognize the foreign body, activate, and proliferate [61]. The utilization of mRNA LNPs as vaccines through the induction of memory CD8^+^ T cells has been explored in multiple studies, where therapeutic mRNAs are delivered to nonspecific immune cells which then present to CD8+ T cells, which further expands antigen‐specific CD8^+^ T cells by priming with APCs. In a report published by Knudson et al. [62], LNPs were delivered *in vivo* through intramuscular injections to APCs encapsulating N1‐methylpseudouridine‐modified ectromelia virus (ECTV) mRNA, which induced a strong CD8^+^ T cell response to the corresponding epitope by priming naïve antigen‐specific CD8^+^ T cells to become protective memory T cells. This was observed through the re‐introduction of the ectromelia virus in an *in vivo* model and the platform facilitated protection of mice from the lethal ECTV challenge. Additionally, the activation of memory CD8+ T cells by the introduction of a specific gene in ECTV provided an inflammatory response from the virus itself, further demonstrating the promise of LNP‐mRNA vaccines in combating other viruses.

Moreover, the delivery of mRNA LNPs has a promising future as vaccines for cancer. A study by Chen et al. [63] delivered ovalbumin, or OVA — a model immunological protein — mRNA to the lymph nodes specifically to induce a stronger CD8^+^ T cell response when introduced to the OVA‐expressing cancer cells. This was observed through an *in vivo* tumor model with B16F10‐OVA cells, where CD8^+^ T cells recognized the tumor cells with the OVA antigen and shifted the immune cell composition to increase the CD8^+^ T cell population and enhance the recruitment of macrophages and major histocompatibility complex (MHC) II^+^ dendritic cells. This shift in immunity resulted in the attack and subsequent killing of cancer cells within the cell population. Another exploration of T cell activation, in addition to CD8^+^ T cell activation, is the use of antibody‐targeted LNPs to CD3^+^ T cells with anti‐CD3 receptors that deliver various mRNAs. As explored in a study by Kheirolomoom et al. [64], the aCD3‐LNPs both activated and transfected CD3+ T cells, which could further activate CD8^+^ and CD4^+^ T cells for downstream immune responses.

Lastly, the modulation of T cells for immune suppression applications is a new and exciting application of T cell activity. In our group, LNPs containing Foxp3 mRNA have been delivered to CD4^+^ T cells *ex vivo* and result in regulatory T (T‐reg) cell activity [65]. This process explores new possibilities for T cells in autoimmunity and introduces alternative uses for T cell modulation by mRNA via LNPs. Expressing Foxp3 in T‐reg cells is advantageous due to the cell's transient nature as it is not a completely suppressive T cell, and therefore will not lead to as many immunogenic side‐effects such as infection and cancer [66]. In the same vein, a disadvantage of this immunoengineering is the lower duration of the therapeutic effect, as the T‐reg cells have a suppressive window for 2–6 days, so LNP delivery of Fopx3 mRNA cannot have prolonged suppression of the immune system for long‐term autoimmunity. Further, T‐reg cells have only been transfected by Foxp3 mRNA‐LNPs *ex vivo*, and thus the efficacy *in vivo* has yet to be seen and will likely require further optimization.

The use of LNPs for CAR T cells and CD8^+^ T cell recruitment for cancer vaccines, cancer immunotherapies, and other applications are still in preliminary stages but show promise for rapid and potent immunological response. Moreover, the utilization of mRNA to deliver antigens for downstream immunological recognition and response provides an effective and safe route for applications in the future. Further research is needed into the efficacy and long‐term effects of CAR T cell generation through LNP technology, as only preliminary studies have been conducted to determine the overall benefits of these platforms. One shortcoming of CAR T cells, however, is their inability to penetrate the solid tumor environment [67]. Another limitation of mRNA LNPs for CAR T cells is their transience, which leads to lower CAR potency *in vivo*. Subsequent research can be conducted to explore other CAR mRNA cargoes, such as circular RNA or self‐amplifying RNA, which mediate longer duration of expression without genome integration. While further studies are needed to optimize the potency and minimize off‐target effects, LNPs provide an exciting alternative to traditional CAR‐T therapies and vaccines.

## NK cells

Although T cell‐based immunotherapies have been successful and their major safety concerns of cytokine release syndrome and graft‐versus‐host disease can be mitigated through mRNA and LNPs, several clinical challenges remain. For example, T cells must be autologous to prevent host–human leukocyte antigen mismatch. As a result, T cell immunotherapies must be derived from the patient, which entails significant expenses and involves a considerable amount of time to complete. Moreover, T cell therapies are not conducive to the heterogenous and immunosuppressive tumor microenvironment (TME) of solid tumors [68].

Thus, NK cells are being studied as an alternative immunotherapy. NK cells, an integral part of the innate immune system, swiftly detect and eliminate infected or aberrant cells through their diverse receptors, playing a crucial role in immune surveillance against viruses and cancer without the need for prior sensitization. As such, NK cells offer versatile and rapid immunotherapeutic potential due to their nonspecific targeting, minimal risk of graft‐versus‐host disease, adaptability, and synergy with other treatments [69, 70]. Their ability to efficiently target a wide range of cancerous cells with low toxicity makes them a promising candidate, even for solid tumors [71]. Furthermore, as part of the innate immune system, NK cell therapies can be sourced allogenically to develop as off‐the‐shelf [72].

Despite these advantages, safe and efficacious NK cell modulation is notoriously difficult because of the complexity of regulating their activity without compromising the body's natural immune surveillance. This includes even the NK‐92 cell line, derived from a patient with non‐Hodgkin's lymphoma that is extensively used in research [73]. Retroviral transduction yields efficiencies of up to 98% and about 50% [74] and lentiviral transduction yields efficiencies of 98% and 30–40% [75] for NK‐92 cells and primary NK cells, respectively. However, these variable transduction efficacies, the potential necessity for multiple rounds of transduction, viral‐associated cell death, and the requirement for posttransduction enrichment may collectively hinder the clinical viability of viral transduction [76]. Similarly, although EP has transfection efficiencies of 80–90% [77], and is safer than viral transduction, it can alter gene expression as well as damage the NK cell membrane, leading to cell death, and making clinical translation challenging [78]. For example, a study by Ingegnere et al. [79] optimized a protocol for EP transfection into NK cells and found that the highest cell viability they could achieve was around 50%.

As such, LNPs are being employed as an alternative delivery vehicle. The initial studies with NK cells were performed with the NK‐92 cell line using a platform similar to modern LNPs to deliver siRNA into NK cells. Nakamura et al. [80] used a multifunctional envelope‐type nanodevice, a pure lipid system that resulted in a 60% and 100% transfection efficiency for a 10 nM and 30 nM siRNA dose, respectively. They also achieved a maximum gene silencing efficiency of 75% for NK‐92 cells compared with 19% from Lipofectamine. However, the platform only induced 55% gene silencing at a siRNA dose that was not cytotoxic [80]. Later, the team introduced a new siRNA core, formed through electrostatic interactions with a protamine polycation into the YSK12‐ multifunctional envelope‐type nanodevice, significantly decreasing cytotoxicity while maintaining gene silencing efficiency. In fact, silencing activity per cellular uptake efficiency and hemolytic activity was more than doubled compared with before [81]. Later studies employed modern LNPs with nonlipid components for mRNA delivery into NK cells. Nakamura et al. [82] developed a CL1H6‐LNP, which achieved a 10‒100× higher eGFP mRNA expression intensity compared with their benchmark, MC3. While both LNPs had 100% cell transfection efficiencies at high mRNA doses of 0.4 µg/mL, CL1H6 had higher efficiencies at lower doses, only dropping to 80% at a 0.066 µg/mL dose compared with 60% by MC3. Cell viability for the CL1H6 was 70% for doses at 0.2 µg/mL and lower with 40% viability at a higher dose of 0.5 µg/mL [82]. Douka et al. [83] found similar results, where their LNPs, made using DSPC and SM‐102, the latter being the ionizable lipid utilized in the Moderna COVID‐19 vaccines, resulted in a better transfection efficiency and higher overall eGFP expression versus EP.

More recently, other studies found success with CAR mRNA cargos. Vital et al. [84] demonstrated the 99% encapsulation efficiency of BCMA or CD19‐CAR with LNPs. Moreover, BCMA‐CAR expression was maintained for 93% and 70% of cells post‐thaw, for 24 and 48 h, respectively, and the CD19‐CAR‐NK cells successfully killed CD19‐positive Raji and Daudi target cells *in vitro* [84]. While there have not been many other studies using LNPs for CAR‐NK cells, success has also been found with LNP‐like nanoparticle drug delivery systems, indicating likely success for future LNP studies. Kim et al. developed multifunctional nanoparticles (MF‐NPs) designed for genetic manipulation and *in vivo* tracking. The MF‐NPs featured a core‐shell structure with a cationic polymer labeled with a near‐infrared fluorescent molecule and a polydopamine coating layer. When applied to NK cells, the MF‐NPs were found to be biocompatible, efficiently delivered genetic materials into the cells, and induced target protein expression, including EGFR targeting chimeric antigen receptors. This genetic modification enhanced the anti‐cancer activity of NK cells *in vitro* and *in vivo*, and the MF‐NP‐labeled NK cells could be successfully tracked using noninvasive magnetic resonance and fluorescence optical imaging [85].

Nevertheless, there are several barriers to LNP‐mediated mRNA delivery in NK cell lines. Most notably, many top‐performing LNPs remain cytotoxic. Nakamura et al.’s CL1H6‐LNP, for example, was derived from the YSK12‐C4 lipid that was designed to minimize toxicity, as previously described, but remained cytotoxic. While better than EP, the cytotoxicity was still greater than desired, especially since only low mRNA concentrations yield less cytotoxicity, resulting in sub 80% transfection efficiencies and lower mRNA expression intensities. Also, while BCMA‐CAR expression was 70% after 2 days, this is a significantly shorter therapeutic duration compared with CAR‐NK cells produced through viral transduction. Thus, CAR‐NK cells made through LNPs must be repeatedly infused into the patient or the LNPs must be delivered *in vivo*, the latter presenting a whole new set of challenges.

Moreover, recent clinical trial results have indicated the limitations of the NK‐92 cell line used in existing works, including the ones above. This is because NK‐92 cells are unable to significantly expand *in vivo*, which can decrease their efficacy in targeting cancer cells [86]. Thus, while several Phase I trials have shown the safety and tolerability of these cells, their clinical benefits are not completely clear [87, 88]. For instance, no antitumor response was observed after NK‐92 cell transfusion to replace impaired NK cells in any patients with refractory/relapsed adult acute myeloid leukemia during a Phase I clinical trial (NCT00900809). This will be a problem regardless of the chosen modification strategy whether it is viral transduction or LNP‐mediated.

As a result, there has been growing interest in RNA delivery into primary NK cells, which can expand more readily *in vivo*. Thus, despite their lower transfection efficiencies, there is hope that they will be more clinically relevant. Furthermore, delivery in primary NK cells would be more conducive for combination therapy with T‐cells such as dual CAR‐T and CAR‐NK regimens since CAR‐T cells must be autologous and thus LNPs could be used to target both patient‐derived T and NK‐cells. Still, while there has been success in LNP‐mediated delivery into NK‐92 cells, the same has not been achieved in primary NK cells. Although once thought of as homogenous with limited but focused immune functions, unlike T‐cells, recent studies have revealed the heterogeneity of NK cells, which can have a different combination of activating and inhibitory receptors to form certain classes of cells such as conventional natural killer cells and tissue‐resident natural killer cells [89, 90]. While not examined in NK cells, studies have confirmed that cell heterogeneity influences LNP‐mediated mRNA delivery with cell receptors with endogenous proteins likely playing a role [91]. This is supported by the significant donor‐to‐donor variability in peripheral blood and cord blood‐derived CAR‐NK cells, which is also true for T cells [92].

Nevertheless, there have been a few studies that have had some promising results. Wilk et al. utilized charge‐altering releasable transporters (CARTs) for mRNA delivery. CARTs are very similar to LNPs as their polymer resembles a lipid polymer, and like LNPs, they become positively charged only at low pH. Compared with EP, CARTs were more efficient at transfecting NK cells, maintained cell viability, and induced minimal alterations to NK‐cell phenotype and function. Furthermore, they were able to generate cytotoxic anti‐CD19 CAR NK cells using this platform.

**Figure 3:**
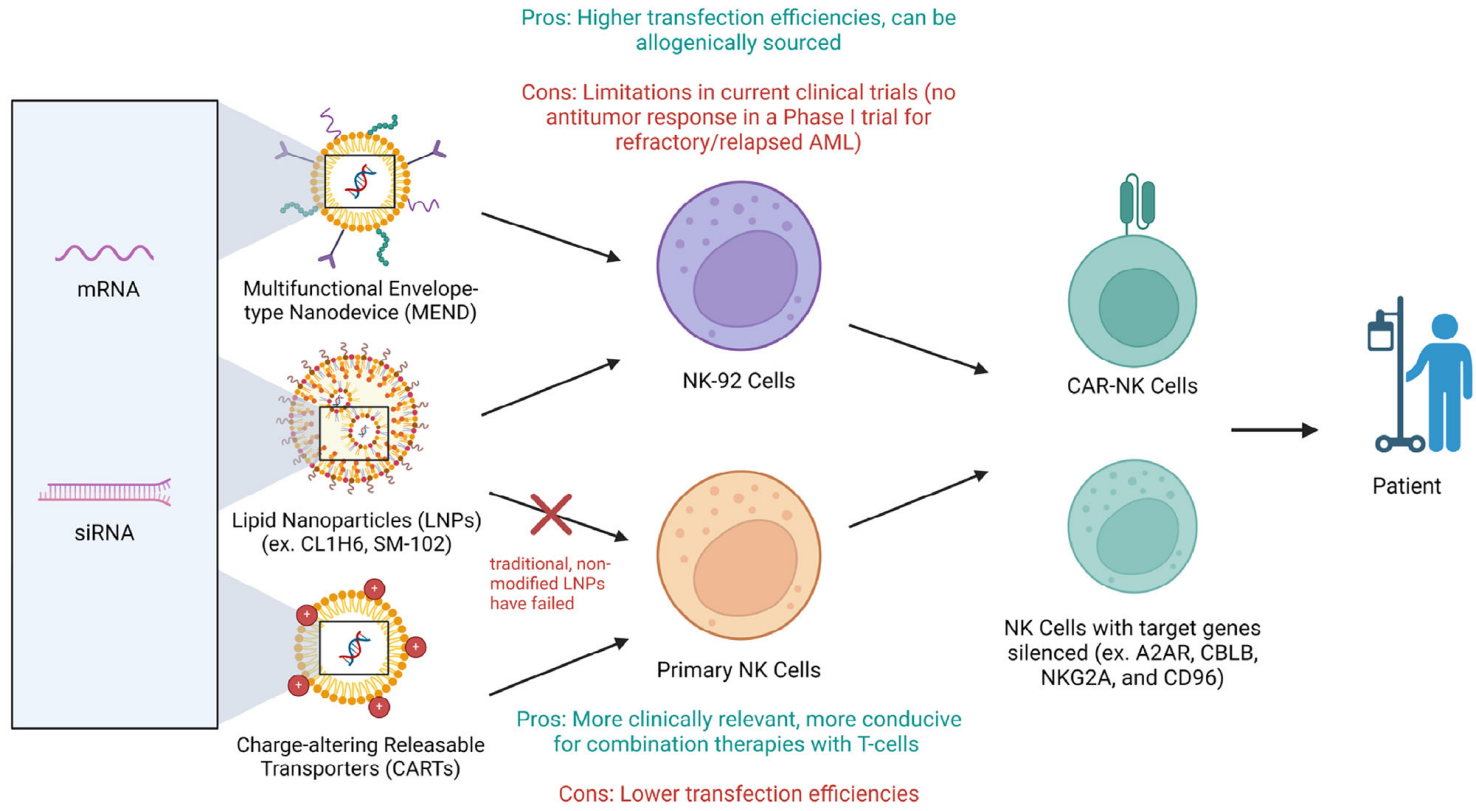
Schematic representation of the different nanoparticles used to modify NK‐92 and primary NK cells and their limitations and desired effects. Created with BioRender.

NK cells are emerging as a promising alternative to T cells due to their adaptability and ability to target various cancer cells with low toxicity. However, transfecting NK cells efficiently and with low toxicity remains a hurdle. While viral methods and EP pose limitations, LNPs show promise in delivering genetic material, especially in NK‐92 cells. However, translating this success to primary NK cells has proven challenging. Since CARTs have shown promise in mRNA delivery to primary NK cells, they could be modulated in an LNP‐like system to further enhance delivery. Overall, while hurdles persist, research into efficient genetic material delivery to NK cells through different methods as described in Fig. 3 offers hope for enhancing immunotherapy effectiveness.

## Macrophages

Macrophages exhibit a complex dual role in cancer, acting both as defenders and enablers of tumor growth [93]. Although various immune cells exhibit functional plasticity, macrophages are uniquely pivotal in cancer due to their direct involvement in tumor progression via their polarization into distinct pro‐inflammatory M1 and anti‐inflammatory M2 phenotypes [94]. M1 macrophages promote antitumoral effects by producing inflammatory cytokines, such as IL‐12 and TNF‐alpha, enhancing antigen presentation and stimulating Th1 immune responses, which collectively contribute to the destruction of tumor cells [95]. Conversely, M2 macrophages support tumor growth and metastasis by producing anti‐inflammatory cytokines like IL‐10 and TGF‐beta, which suppress adaptive immune responses and promote tissue remodeling, angiogenesis, and tumor cell invasion. This dual ability underscores their significance as key targets in cancer immunotherapy, where manipulating these phenotypes can directly alter the TME [96].

Leveraging this duality, researchers have adapted CAR technology to macrophages, creating CAR‐macrophages (CAR‐Ms). Unlike CAR‐T cells, primarily used in hematologic malignancies, CAR‐Ms demonstrate unique capabilities in solid tumor environments by targeting and destroying cancer cells displaying specific antigens in the TME [97]. A pivotal study by Klichinsky et al. [98] demonstrated that CAR‐Ms could phagocytose and destroy tumor cells, showing significant tumor reduction in mouse models of ovarian cancer. However, the development of CAR‐Ms faces challenges, particularly in genetic engineering, due to the innate resistance of primary macrophages to viral vectors used for gene delivery. LNPs have emerged as a safer and more efficient alternative for delivering mRNA to macrophages, facilitating the creation of CAR‐Ms capable of targeting solid tumors effectively. Modulating non‐CAR macrophages, especially tumor‐associated macrophages (TAMs), presents another viable therapeutic pathway. Vitamin C, known for its antioxidant properties, can induce oxidative stress in cells, selectively killing cancer cells without harming normal cells and promoting the polarization of macrophages from the M2 phenotype to the immunostimulatory M1 phenotype. Ma et al. utilized LNPs to encapsulate vitamin C, ensuring its controlled release directly at the tumor site [99]. This approach induced oxidative stress and promoted the polarization of macrophages from the M2 to the M1 phenotype, as evidenced by increased CD80 and decreased CD206 expression, along with enhanced T‐cell responses in vitro and murine models of melanoma. Similarly, Gao et al. [100] developed an injectable hydrogel loaded with immune regulatory LNPs containing mRNA and siRNA to upregulate immune regulatory factor 5 and downregulate C−C chemokine ligand 5. This hydrogel system demonstrated significant TAM reprogramming and tumor growth inhibition in both *in vitro* studies with RAW 264.7 macrophages and *in vivo* studies using a pancreatic cancer model.

Improving targeted LNP delivery to macrophages is crucial for enhancing the effectiveness of therapies that rely on macrophage modulation. Efficient delivery ensures that a higher proportion of therapeutic agents reach the target cells, amplifying therapeutic effects while minimizing off‐target impacts. Targeting the sigma‐1 receptor (Sigma‐1R) for the delivery of the relaxin gene using conjugated lipid‐DNA nanoparticles has shown promise. Sigma‐1R is predominantly expressed on several cell types within the TME, including macrophages and fibroblasts, and targeting this receptor facilitated the reprogramming of macrophages from an M2‐like to a more proinflammatory M1‐like state [101]. Zhou et al. [102] utilized the KPC mouse model of pancreatic ductal adenocarcinoma to explore this approach, achieving significant tumor reduction and enhanced T‐cell infiltration. In contrast, Rafique et al. focused on using calcitriol embedded in LNPs targeting the macrophage‐specific endocytic scavenger receptor CD163 [103]. Their approach demonstrated enhanced uptake by macrophages, effective inhibition of proinflammatory markers, and upregulation of IL‐10 mRNA gene expression in human monocyte‐derived macrophages and ex vivo mouse tissue models, illustrating a highly selective mechanism of delivery.

Combination therapies that integrate LNP‐based therapies with chemotherapy or radiotherapy could further enhance the therapeutic efficacy of macrophage‐based medicines. Such combination approaches exploit the strengths of each modality, potentially leading to synergistic effects where the combined therapeutic impact is greater than the sum of the individual effects. Zhang et al. developed an innovative terpolymer‐lipid hybrid nanoparticle system capable of crossing the blood–brain barrier to target tumor cells and TAMs [104]. This system utilized the iRGD peptide, which enhances drug penetration into tumor tissues by binding to αvβ3 and αvβ5 integrins overexpressed in tumors. The study demonstrated a significant reduction in metastatic burden and TAM populations in the brain in murine models of triple‐negative breast cancer brain metastases. These findings highlight the potential of LNPs for precise delivery of therapeutic agents to macrophages at challenging target sites, overcoming physiological barriers like the blood–brain barrier, and presenting new possibilities for treating inflammation‐related diseases and cancers.

While the studies reviewed provide valuable insights into the potential of LNPs and CAR‐Ms in cancer immunotherapy, several limitations are evident. Many studies solely utilize murine models that do not fully replicate human tumors or lack a competent immune system, limiting translatability. This is furthered by the difficulty in genetic engineering of primary macrophages via vectors to establish more relevant models. Moreover, LNP delivery studies, such as those targeting the sigma‐1 receptor and using calcitriol, demonstrated efficacy but were limited using genetically engineered mouse models and *ex vivo* studies, which do not accurately predict clinical outcomes. From the LNP perspective, enhancing mRNA internalization and endosomal escape, and optimizing formulation parameters like hydrophobicity and fusogenicity, are crucial. Adding targeting moieties such as CD163 and integrating LNP therapies with chemotherapy or radiotherapy could enhance efficacy. Addressing these issues requires focused research to refine formulations and explore innovative cancer treatment strategies, aiming for more effective, targeted, and personalized therapies.

## Dendritic cells

Dendritic cells (DCs) are potent antigen‐presenting cells that play a significant role in activating memory T cells, as DCs are involved in both differentiating naïve T cells and generating memory T cells. Dendritic cells possess features that characterize them as APCs [105]. When a DC encounters an antigen, it processes and presents it on its surface in association with MHC molecules. This antigen presentation, along with co‐stimulatory signals provided by the dendritic cell, activates naïve T cells. Dendritic cells can also stimulate the generation of memory cells [106]. During the activation of naïve T cells, some differentiate further into memory T cells.

As such, DC therapy aims to stimulate T cells without over‐activating or inducing toxicity [107]. DCs have been in clinical use for over three decades with ongoing clinical trials for immunotherapies [108]. Since they are major cytotoxic and humoral adaptive response regulators, DCs are commonly employed as targets for mRNA‐based vaccine therapies as they can be stimulated to present antigens to T‐cells [109]. Additionally, DCs can be modified to carry antigens that are typically whole proteins or peptide fragments, which are processed and presented on MHCs to then activate T cells [110].

Although mRNA‐based DC vaccines have high therapeutic potential, delivery into the DC cytosol has been challenging. This is because DCs are very selective in which molecules cross their membranes, making them difficult to transfect. Although viral methods have been established to effectively transfect DCs, alternative nonviral methods, including the use of LNPs for effective transfection, are still being analyzed [111]. For example, LNPs have been employed to deliver the nucleic acid adjuvant polyinosinic:polycytidylic acid, which was shown to activate DCs and facilitate their migration to the lymph nodes [112]. LNPs have also been used to deliver siRNA to dendritic cells, inducing a 75% knockdown in CD80 expression [113]. Here these LNPs were coated with a single‐chain antibody scFv specific to murine DEC205, which is highly expressed on some DC subsets. The LNPs were injected intravenously in B6 mice, where the mixed lymphocyte response was inhibited by the gene knockdown. These results underscore the vast potential for LNPs as a therapeutic for various autoimmune diseases through genetic modification.

Sasaki et al. [114] analyzed the *in vivo* targeted transfection of splenic DCs by characterizing a library of 8 different LNPs. The researchers showcased that high ionizable lipid percentage, lower %PEG, higher NaCl concentration, ζ‐average, and size were all significant factors affecting cellular uptake. For instance, cellular uptake sharply increased with ζ‐average for particles up to approximately 200 nm. The A‐11‐LNP formulation had the greatest transgene activity and was compared against two clinically relevant LNP formulations: MC3‐LNP, the first‐ever developed siRNA therapeutic for transthyretin amyloidosis targeting liver tissue, and RNA‐LPX, a formulation developed to delivery mRNA specifically into splenic DCs. At mRNA dosages of 0.5 mg/kg, the A‐11‐LNPs resulted in approximately 9% of EGFP+ DCs, showing a much higher transgene expression than the two other LNP formulations and therefore higher potential for mRNA vaccine applications.

Furthermore, to increase its effectiveness in promoting anti‐cancer immunity, DC therapy is often combined with other approaches, including monoclonal antibodies, chemotherapy, cytokine‐induced killer cells, and radiotherapy [115]. In addition to providing an alternative to toxic conditioning protocols in stem cell transplantation, LNPs have also been used to modify the cellular environment in order to enhance DC therapy *in vivo* [116]. For instance, LNPs carrying mRNA can induce cell death in tumoral tissues, resulting in the expression of the CD40 ligand, and activating dendritic cells that were previously genetically modified by the LNPs with CD40 mRNA. The dendritic cells then present the tumor‐associated antigens to T cells and induce tumor‐specific T‐cell immunity. Dendritic cell therapy is a therapeutic platform with significant potential to overcome limitations of other methods such as adverse TME and low tumor cytotoxicity; LNPs are a multi‐faceted therapeutic platform that can help improve genetic modification and vaccine delivery in DCs.

## Stem cells

**Figure 4:**
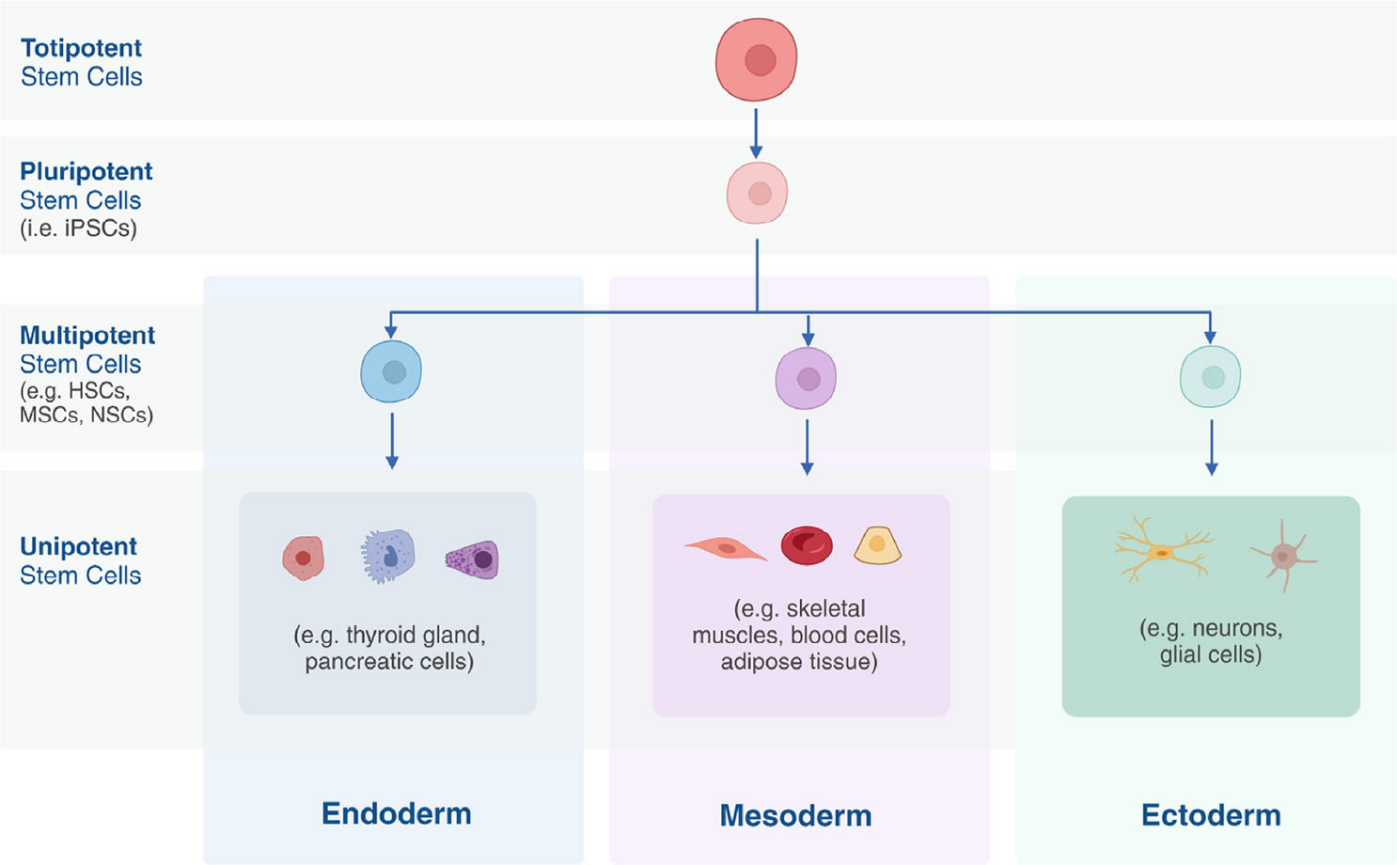
Differentiation of totipotent stem cells into endoderm, mesoderm, or ectoderm cells. Created with BioRender.

Although stem cells are not considered directly part of the immune system, they play a vital role in supporting its functions. Stem cells are undifferentiated cells with the potential to develop into various specialized cell types, including those involved in immune responses. For instance, hematopoietic stem cells (HSCs), a type of multipotent stem cell found in the bone marrow, differentiate into all blood cells, including white blood cells that are central to the immune system as seen in Fig. 4. These white blood cells, such as lymphocytes, monocytes, and neutrophils, are responsible for recognizing and combating foreign invaders like pathogens and cancer cells. HSCs also have the capability to differentiate into various types of blood cells, including T cells, B cells, and other immune cells [117].

As a result, stem cells have significant potential for both *ex vivo* and *in vivo* immunotherapies as they can enhance cell populations to promote tissue homeostasis and regeneration via their self‐renewal and differentiation properties [118]. Stem cell‐based treatments boost the body's natural repair processes by stimulating, adjusting, and overseeing the existing stem cell population to facilitate regeneration. Therefore, they have been used to facilitate transplantation by mitigating rejection rates. Numerous strategies have been developed to optimize these therapies [119]. Human pluripotent stem cells, HSCs, and multipotent mesenchymal stem cells (MSCs) have been used in new key cellular therapies to treat human diseases such as neurological and cardiovascular conditions, including multiple sclerosis and ischemic heart diseases [120]. MSCs have also been as new therapeutic tool for acute kidney injuries [121].

Despite the advantages of stem cells, the self‐renewal property of these cells is limited *in vitro*. Although stem cells can be transfected via lipofectamine or EP with nucleic acids to induce cell division, these methods have therapeutic limitations. Lipofectamine is typically regarded as the “gold standard” for delivering exogenous DNA or RNA into cells [122]; however, although recent studies have tested *in vivo*, its efficacy is limited *in vivo* due to its poor targeting and high toxicity.

While the efficiency of lipofectamine is dose‐dependent, lipofectamine can be toxic at higher concentrations [123]. On the other hand, EP typically achieves a higher transfection rate but induces higher cell toxicity. EP can result in various cell injuries leading to cell death, including membrane damage, ATP depletion, protein damage, and mitochondrial damage [124]. For instance, in a study transfecting dental pulp stem cells, EP induced 63% transfection efficiency using a 100 V, 20 ms, one‐pulse square‐wave condition, while 1:1 DNA:Lipofectamine transfected up to 19% of cells [125]. Currently, both lipofectamine and EP are limited to *in vitro* applications due to concerns regarding systemic toxicity. A study comparing formulations’ transfection efficiencies after injection into developing chick embryos noted that concentrated 1:1 Lipofectamine 2000 quickly formed inactive aggregates, becoming unusable within 20 min as precipitates formed and transfection efficiency significantly dropped [126]. Therefore, LNPs, which have demonstrated high efficiency in *in vivo* applications, are becoming increasingly more validated and can be a comparable transfection method for both *ex vivo* and *in vivo* applications to overcome these limitations of Lipofectamine and EP.

For example, LNPs can be utilized to facilitate MSC differentiation to prepare them for bone marrow transplantation, which is a procedure that can restore innate and adaptive immune cell populations [127, 128]. In one study, LNPs containing siRNA to silence the suppressor gene GNAS were employed to facilitate osteogenic differentiation of MSCs *in vitro*. Since previous studies have found LNPs formulated with 1,2‐dioleoyl‐3‐trimethylammonium‐propane, a permanently charged cationic lipid, to have limited efficacy in non‐hepatic tissues, the LNPs in this study were formulated with 1.5 mol% distearoyl‐rac‐glycerol‐PEG2K (DSG‐PEG2000), which has a longer residence time in the LNP particle and therefore lengthens circulation time and improves distribution to the bone. However, the residence time was still under 15 min, requiring an increased dosage to achieve distribution to the bone, indicating further optimization for target specificity must be analyzed [129]. Despite the short residence time, the formulated LNP significantly reduced GNAS levels by approximately 80%, 7 days after treating the cells. These results indicate that LNPs can be used to induce genetic modification of the MSCs of the bone marrow. While this work is specific to MSCs, the trafficking and delivery of LNPs to the bone marrow opens the possibility for delivery to other bone marrow resident cells, such as HSCs, which differentiate into myeloid and lymphoid progenitor cells.

To that end, there have been recent studies using LNPs for HSC therapies, as current HSC therapies require toxic conditioning protocols such as chemotherapy in order for the body to adapt to the endogenous HSCs; these protocols help deplete unhealthy cells before new cells are transplanted [130]. LNPs provide an alternative method to these toxic conditioning protocols by inducing gene modification and editing in HSCs. In addition to modifying the cargo, LNPs can also be modified to target specific oncogene receptors by conjugating antibodies to the LNP [131]. In one study, an LNP encapsulating Cre mRNA was employed to modify HSCs *ex vivo* by attaching an antibody targeting stem cell factor receptor CD117 [132]. The CD117‐targeting LNP demonstrated 95% *ex vivo* genome editing in LinSca‐1c‐kit cells, a subfraction of bone marrow cells that contain all multipotent hematopoietic cells, higher than the nontargeted control LNP. *In vivo*, this LNP‐mediated mRNA transfection in 64% of bone marrow cells was successfully used to facilitate bone marrow transplantation by delivering proapoptotic PUMA mRNA. Another study designed a similar antibody‐conjugated CD117‐targeting LNP to deliver mRNA to HSCs in vivo [133]. Here, the LNPs were formulated with a functionalized maleimide PEG‐lipid to conjugate the CD117 antibody, whereas up to 90% of the HSCs were transfected in a dose‐responsive manner. These studies demonstrate the extraordinary efficacy and targeting abilities of LNPs for stem cell therapies.

An important aspect is that the lipofection of stem cells typically entails the delivery of Cas9 protein rather than Cas9 mRNA. Although LNPs have the capability to deliver proteins, challenges in encapsulation and efficacy persist. According to a study conducted by Walther et al. [134], protein‐loaded LNPs were approximately 100 nm larger than mRNA‐loaded LNPs, posing difficulties in transfection with low toxicity. However, in methods such as HDR, the use of proteins offers advantages as it enables simultaneous cleavage of the two target genes, unlike mRNA, which requires time for translation into protein before cleavage, potentially leading to timing discrepancies. Many aspects of LNPs still await further improvements.

Stem cell therapies demonstrate significant potential as therapeutic platforms for treating conditions by regenerating and replacing unhealthy cells with healthy cells. LNPs take these therapies one step further by facilitating therapeutic mRNA delivery while minimizing toxicity. As such, LNPs can help enable the production of healthy or even modulated immune cells that then have the potential for downstream therapeutic effects. In particular, the successful utilization of LNPs to target bone marrow presents a promising opportunity to tackle a range of diseases including lymphomas, aplastic anemia, immune deficiencies, and leukemia. Furthermore, while there is some research in adult stem cells, embryonic stem cell therapies also have shown potential since embryonic stem cells can differentiate into any of the 200 cell types in the human body whereas adult stem cells are limited. Stem cell therapies present a promising therapeutic option, especially with the advancements in LNP technology (Table 1).

**Table 1:** Ongoing/completed clinical trials for listed cell therapies.

	Application	ID
Dendritic cell vaccine in treating patients with indolent B‐cell lymphoma or multiple myeloma	Lymphoma, multiple myeloma and plasma cell neoplasm	NCT00937183
Pembrolizumab and autologous dendritic cells for the treatment of refractory colorectal cancer (CRC)	Colorectal Cancer	NCT05518032
Tumor lysate‐pulsed dendritic cell immunotherapy for patients with brain tumors	Glioblastoma	NCT00576537
Allogeneic stem cells’ implantation combined with coronary bypass grafting in patients with ischemic cardiomyopathy	Coronary artery disease, ischemic cardiomyopathy	NCT01753440
Allogenic adipose tissue‐derived mesenchymal progenitor cells for the treatment of knee osteoarthritis	Knee osteoarthritis	NCT04208646
Phase 1 clinical trial to evaluate the safety of allogeneic NK Cell (“SMT‐NK”) cell therapy in advanced biliary tract cancer	Advanced biliary tract cancer	NCT03358849
High‐activity natural killer immunotherapy for small metastases of melanoma	Melanoma	NCT03007823
High‐activity natural killer immunotherapy for small metastases of colorectal cancer	Metastatic colorectal cancer	NCT03008499

*Note*: Trials found on ClinicalTrials.gov, created under the NIH National Library of Medicine.

## Conclusion

Progress in immunoengineering has improved due to the remarkable advancements in nanotechnology. This includes LNPs, which have tremendous potential for the development of immunotherapies due to their potency, low toxicity, and versatility, the latter demonstrated by their use in T cells, NK cells, macrophages, stem cells, and dendritic cells. Moreover, LNPs have enabled the use of more complex nucleic acid‐based tools, especially mRNA and siRNAs. Likely, emerging RNA technologies, such as circular and self‐amplifying RNAs will allow for longer‐lasting therapies. Additionally, the utilization of Cas9, or other gene editing mRNAs, will foster the rise of precision gene editing immunotherapies, opening a wide range of possible therapeutic applications.

However, despite the enormous potential of LNPs as a therapeutic platform, there are still hurdles that need to be overcome. While numerous modifications to the four LNP components have been performed, structure‐function studies are lacking, meaning newly developed lipid excipients are tested using trial and error. By examining the role of molecular parameters, such as ionic charge, lipid length, and steroid architecture, as well as particle physiochemical parameters, including size, ζ‐potential, relative p*K*_a_, and morphology, lipids could be generated based on rationale design. Moreover, many lipids, particularly ionizable lipids, are inherently inflammatory, which may be beneficial for certain therapies, but more often, is a disadvantage. Installing more readily degradable moieties could be one route for less immunogenic lipids. Moving forward, a systematic understanding of the structure‐function relationships of LNPs is crucial to improving rational design and optimization. Strategies to mitigate the inherent immunogenicity will be pivotal in realizing the full therapeutic potential of LNPs. Lastly, targeting of specific immune populations has been achieved, but in a limited capacity. While antibodies are an excellent tool for active targeting, they may also induce their own off‐target immune response. Clever manipulation of antibodies, including utilizing antibody fragments or affibodies are possible pathways, as is the use of peptides or aptamers.

In recent decades, LNPs have emerged as a powerful immunoengineering platform, capable of functioning as vaccines and targeting diseases like glioblastoma and cardiac fibrosis. With the ability to silence, mimic, and edit genes utilizing RNAs, LNPs are ushering in a new era of immunotherapy.

## Conflicts of interest

The authors declare no financial and commercial conflict of interest.

## Author contributions

Emily H. Kim, Sridatta V. Teerdhala, Marshall S. Padilla, Ryann A. Joseph, and Jacqueline J. Li wrote the manuscript. Emily H. Kim and Ryann A. Joseph prepared figures. Rebecca M. Haley and Michael J. Mitchell revised the manuscript.

**Abbreviations**
aCD19: anti‐CD19
APCs: antigen‐presenting cells
CAR: chimeric antigen receptor
CARTs: charge‐altering releasable transporters
CDs: clusters of differentiation
DCs: dendritic cells
ECTV: ectromelia virus
HSCs: hematopoietic stem cells
LNPs: lipid nanoparticles
MF‐NPs: multifunctional nanoparticles
MSCs: mesenchymal stem cells
TAMs: tumor‐associated macrophages
TME: tumor microenvironment

## Data Availability

No new data were created or utilized for this review.
